# A novel mutation in the connexin 46 (*GJA3*) gene associated with congenital cataract in a Chinese pedigree

**Published:** 2011-05-20

**Authors:** Xuchen Ding, Binbin Wang, Yongfeng Luo, Shanshan Hu, Guangkai Zhou, Zhou Zhou, Jing Wang, Xu Ma, Yanhua Qi

**Affiliations:** 1 Department of Ophthalmology, The Second Affiliated Hospital of Harbin Medical University, Harbin, China; 2 Department of Genetics, National Research Institute for Family Planning, Beijing, China

## Abstract

**Purpose:**

To identify the potential pathogenic mutation in a three-generation Chinese family with congenital nuclear pulverulent cataracts.

**Methods:**

A three-generation pedigree was recruited for our study. Three patients and four healthy members of the family underwent a comprehensive clinical examination. Genomic DNA extracted from peripheral blood was amplified using the polymerase chain reaction (PCR) method and the exons of all candidate genes were sequenced.

**Results:**

When sequencing the encoding regions of the candidate genes, a novel mutation (c.559C>T) was identified in the gap junction protein alpha 3 (*GJA3*) gene, which resulted in the substitution of highly conserved proline by serine at codon 187 (P187S). There was no noticeable nucleotide polymorphism in other candidate genes. The mutation co-segregated with all patients, but was absent in the healthy members and 100 normal individuals.

**Conclusions:**

The present study identified a novel mutation (c.559C>T) in the *GJA3* gene associated with autosomal dominant pulverulent cataracts in a Chinese family. As the first report to relate p.P187S mutation in *GJA3*, it expands the mutation spectrum of *GJA3* in association with congenital cataracts.

## Introduction

Cataracts are the most common cause of visual impairment in the world. They are especially evident in blindness in infants, as it is reported that approximately 10% of all childhood blindness worldwide is due to congenital cataracts [1]. Congenital cataracts have been reported primarily as an autosomal dominant disorder and rarely as an autosomal recessive or X-linked pattern. To date, the genes that have been identified in congenital cataracts include ten crystalline genes (αA-crystallin [*CRYAA*] [2], αB-crystallin [*CRYAB*] [3], βA1-crystallin [*CRYBA1*] [4], βA4-crystallin [*CRYBA4*] [5], βB1-crystallin [*CRYBB1*] [6], βB2-crystallin [*CRYBB2*] [7], βB3-crystallin [*CRYBB3*] [8], γC-crystallin [*CRYGC*] [9], γD-crystallin [*CRYGD*] [10], and γS-crystallin [*CRYGS*] [11]), two cytoskeletal protein genes (beaded filament structural protein 2, phakinin [*BFSP2*] [12], beaded filament structural protein 1, filensin [*BFSP1*] [13]), three transcription factors genes (heat shock transcription factor 4 [*HSF4*] [14], Maf-like protein [*MAF*] [15] and paired-like homeodomain 3 [*PITX3*] [16]), Glucosaminyl (N-acetyl) transferase 2 (*GCNT2*) [17], chromatin-modifying protein-4B (*CHMP4B*) [18], transmembrane protein 114 (*TMEM114*) [19], and four membrane transport protein genes (major intrinsic protein of lens fiber [*MIP*] [20], lens intrinsic membrane protein 2 gene [*LIM2*] [21], gap junction protein [alpha 8, *GJA8*] [22] and gap junction protein [alpha 3, *GJA3*] [23]).

To date, several mutations in genes have been reported as being associated with congenital nuclear pulverulent cataracts such as *MIP* [24],*CRYBB1* [25], *CRYBA1* [26], *GJA8* [27], and *GJA3* [28]. These mutations had all been identified as a pathogenic mutation. The gap junction protein alpha 3 (*GJA3*) gene consists of a single exon encoding a 435 amino acid protein in humans and is predominantly expressed in lens fiber cells. In the human eyes, three members of the connexin family, including connexin 43 (Cx43), connexin 46 (Cx46), and connexin 50 (Cx50) are expressed. Connexins are the critical function groups on the gap junction channels that play an irreplaceable role in the transportation of metabolites between cells [29], such as ions, metabolites, signaling molecules, and other molecules with a molecular weight up to 1 kDa.

In this study, we detected a novel mutation (P187S) in *GJA3* associated with autosomal dominant nuclear pulverulent cataracts in a Chinese family. The mutation was co-segregated with all patients, but was absent in the healthy members and 100 normal individuals. This mutation has not been reported previously in terms of congenital cataracts, thus knowledge of the mutation will help in understanding the mechanisms of cataractogenesis.

## Methods

### Patient ascertainment and clinical examination

**Figure 1:**
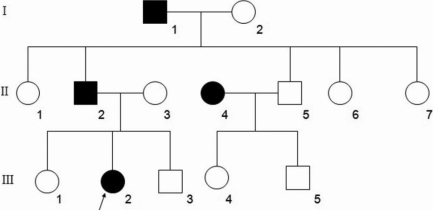
Pedigree of the family. Pedigree of the family with four affected individuals: the proband (III:2), her father (II:2), grandfather (I:1), and aunt (II:4). Circles represent females, while squares indicate males. Shaded shapes indicate affected individuals. The arrow points to the proband.

A three-generation Chinese Han family (Figure 1) with autosomal dominant pulverulent cataracts from Heilongjiang province was identified. Seven members of the pedigree were involved in this study. The research was approved by the Institutional Review Board of Harbin Medical University. The affected and unaffected individuals in this family were accepted full ophthalmic and clinical examinations. In addition, 100 healthy normal were recruited as the normal controls. Photographs of significant findings were taken. After informed consent, venous blood (5 ml) was collected for genomic DNA extraction. Briefly, 5 ml of venous blood from family members and controls was collected in a BD Vacutainer (BD, San Jose, CA) containing EDTA. Genomic DNA was extracted by QIAamp DNA Blood Mini Kits (QIAGEN Science, Germantown, MD).

### Mutation detection

The coding exons and the flanking regions of all the known candidate genes associated with autosomal dominant congenital nuclear pulverulent cataracts, such as *CRYAA*, *CRYAB*, *CRYBA1*, *CRYBB2*, *CRYGC*, *CRYGD*, *CRYGS*, *GJA3*, and *GJA8*, were amplified by PCR with primers listed in Table 1. Through sequencing both directions with an ABI 3130XL Genetic Analyzer (Applied Biosystems, Foster City, CA), the results were analyzed using Chromas (version 1.0) software and compared with the reference sequences in the NCBI gene bank.

**Table 1:** Oligonucleotide primers used for PCR.

**Exon**	**Forward**	**Reverse**	**Annealing temperature (°C)**	**Product length (bp)**
CRYAA-1	5′-AGCAGCCTTCTTCATGAGC-3′	5′-CAAGACCAGAGTCCATCG-3′	61.4	584
CRYAA-2	5′-GGCAGGTGACCGAAGCATC-3′	5′-GAAGGCATGGTGCAGGTG-3′	61.4	550
CRYAA-3	5′-GCAGCTTCTCTGGCATGG-3′	5′-GGGAAGCAAAGGAAGACAGA-3′	61.4	511
CRYAB-1	5‘-AACCCCTGACATCACCATTC-3′	5′-AAGGACTCTCCCGTCCTAGC-3′	64.7	250
CRYAB-2	5′-CCATCCCATTCCCTTACCTT-3′	5′-GCCTCCAAAGCTGATAGCAC-3′	62	350
CRYAB-3	5′-TCTCTCTGCCTCTTTCCTCA-3′	5′-CCTTGGAGCCCTCTAAATCA-3′	62	400
CRYGC-1	5′-TGCATAAAATCCCCTTACCG-3′	5′-CCTCCCTGTAACCCACATTG-3′	59	514
CRYGC-2	5′-TGGTTGGACAAATTCTGGAAG-3′	5′-CCCACCCCATTCACTTCTTA-3′	59	430
CRYGD-1	5′-CAGCAGCCCTCCTGCTAT-3′	5′-GGGTCCTGACTTGAGGATGT-3′	61.4	550
CRYGD-2	5′-GCTTTTCTTCTCTTTTTATTTCTGG-3′	5′-AAGAAAGACACAAGCAAATCAGT-3′	61.4	308
CRYGS-2	5′-GAAACCATCAATAGCGTCTAAATG-3′	5′-TGAAAAGCGGGTAGGCTAAA-3′	61.4	575
CRYGS-3	5′-AATTAAGCCACCCAGCTCCT-3′	5′-GGGAGTACACAGTCCCCAGA-3′	61.4	479
CRYBA1–1	5′-GGCAGAGGGAGAGCAGAGTG-3′	5′-CACTAGGCAGGAGAACTGGG-3′	62	550
CRYBA1–2	5′-AGTGAGCAGCAGAGCCAGAA-3′	5′-GGTCAGTCACTGCCTTATGG-3′	61.4	508
CRYBA1–3	5′-AAGCACAGAGTCAGACTGAAGT-3′	5′-CCCCTGTCTGAAGGGACCTG-3′	62	463
CRYBA1–4	5′-GTACAGCTCTACTGGGATTG-3′	5′-ACTGATGATAAATAGCATGAACG-3′	62	355
CRYBA1–5	5′-GAATGATAGCCATAGCACTAG-3′	5′-TACCGATACGTATGAAATCTGA-3′	62	597
CRYBA1–6	5′-CATCTCATACCATTGTGTTGAG-3′	5′-CATCTCATACCATTGTGTTGAG-3′	62	528
CRYBB2–1	5′-GTTTGGGGCCAGAGGGGAGTGGT-3′	5′-TGGGCTGGGGAGGGACTTTCAGTA-3′	59	350
CRYBB2–2	5′-CCTTCAGCATCCTTTGGGTTCTCT-3′	5′-GCAGTTCTAAAAGCTTCATCAGTC-3′	59	330
CRYBB2–3	5′-GTAGCCAGGATTCTGCCATAGGAA-3′	5′-GTGCCCTCTGGAGCATTTCATAGT-3′	59	360
CRYBB2–4	5′-GGCCCCCTCACCCATACTCA-3′	5′-CTTCCCTCCTGCCTCAACCTAATC-3′	60.7	230
CRYBB2–5	5′-CTTACCCTTGGGAAGTGGCAATGG-3′	5′-TCAAAGACCCACAGCAGACAAGTT-3′	60.7	600
GJA3–1	5′-CGGTGTTCATGAGCATTTTC-3′	5′-CTCTTCAGCTGCTCCTCCTC-3′	61.4	450
GJA3–2	5′-GAGGAGGAGCAGCTGAAGAG-3′	5′-AGCGGTGTGCGCATAGTAG-3′	61.4	450
GJA3–3	5′-TCGGGTTCCCACCCTACTAT-3′	5′-TATCTGCTGGTGGGAAGTGC-3′	61.4	300
GJA8–1	5′-CCGCGTTAGCAAAAACAGAT-3′	5′-CCTCCATGCGGACGTAGT-3′	61.4	420
GJA8–2	5′-GCAGATCATCTTCGTCTCCA-3′	5′-GGCCACAGACAACATGAACA-3′	61.4	330
GJA8–3	5′-CCACGGAGAAAACCATCTTC-3′	5′-GAGCGTAGGAAGGCAGTGTC-3′	61.4	350
GJA8–4	5′-TCGAGGAGAAGATCAGCACA-3′	5′-GGCTGCTGGCTTTGCTTAG-3′	61.4	500

Summary of the primers, annealing temperatures and product length of the candidate genes related with nuclear cataract.

### Bioinformatics analysis

The wild-type and mutant Cx46 protein sequences were analyzed with PolyPhen to predict whether the amino acid substitution affects the structure and function of proteins.

## Results

### Clinical examination

**Figure 2:**
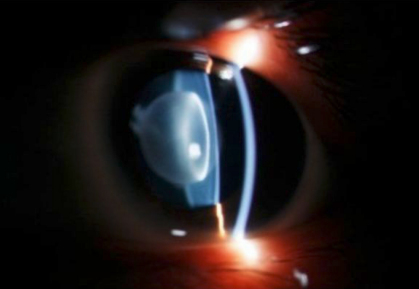
Slit-lamp photograph of the proband. Slit-lamp examination of the proband showed a cataract characterized as a central nuclear opacity with punctate opacities.

There were four affected individuals in this Chinese family, and three of them (II:2, II:4, and III:2) participated in this study (Figure 1). A cataract characterized as a central nuclear opacity with punctate opacities (Figure 2) was exhibited fully in the affected patients. There were no other ocular or systemic abnormalities. To date, all of the affected individuals have had cataract surgery.

### Mutation analysis

**Figure 3:**
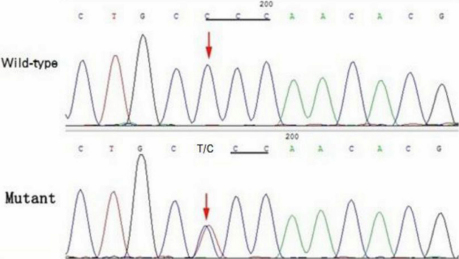
Mutation analysis of the connexin 46 gene (*GJA3*). The sequence chromatogram (forward strand) shows a heterozygous T>C transition that changes proline to serine at codon 187. The red arrows show the wild-type (normal) and mutant point, respectively.

**Figure 4:**
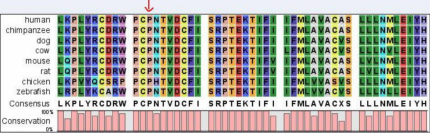
Phylogenetic conservation analysis. The alignment of the GJA3 sequence with the corresponding segments in diverse species was shown. The 187th proline was highly conserved in connexin 46 proteins from several species. The red arrow indicates high conservation.

The candidate genes were sequenced in three affected individuals and four healthy members. A heterozygous missense mutation, C>T at position 559 in exon 2 of *GJA3*, was identified (Figure 3). The mutation led to the substitution of proline by serine, which is highly conserved across various species, as determined from NCBI (Figure 4). There was no noticeable nucleotide polymorphism in the other candidate genes. This change was co-segregated with the affected individuals of the family, but was not detected in either the unaffected family individuals or the 100 normal controls.

### Bioinformatics analysis

PolyPhen analysis demonstrated that the substitution in *GJA3* at position 187 from P to S scored 1.753, which meant that “this variant is predicted to be possibly damaging.”

## Discussion

The *GJA3* gene, which encodes a 435-amino acid protein, is located on chromosome 13q11 and was first reported by Willecke et al. [30], in 1990. Our study identifies a new mutation (c.559C>T) in *GJA3*. This substitution resulted in the replacement of proline by serine at amino acid 187 (P187S). The variant is likely to be disease causative, as it co-segregates with the phenotype of ADCC in all affected individuals in this family, but not with the unaffected individuals and 100 normal controls. As an avascular organ, the eye lens has developed an extensive cell to cell communication system via gap junction channels belonging to connexins that allow the transport of small metabolites between cells. In humans, more than 20 genes that code connexins have been identified. Three of these (connexin 43, connexin 46 and connexin 50) are expressed in the lens. Cx43 is expressed mainly in the lens epithelial cells; Cx46 and Cx50 are expressed in lens fiber cells. Both Cx46 and Cx50 knockout mice develop nuclear cataracts, while deletion of Cx50 also is associated with a significant ocular growth defect [31]. Mutations in either connexin 46 or connexin 50 have been linked with congenital cataracts in humans [32,33].

Like other connexins, *GJA3* has four transmembrane domains (M1, M2, M3, and M4), two extracellular loops (E1 and E2), an intracellular loop (CL), and intracellular NH2 and COOH termini. Our finding is the third mutation detected in E2 of connexin 46 [34,35]. Sequence comparison of connexin 46 showed that the proline at position 187 is phylogenetically conserved in the second transmembrane domain in different species (Figure 4). Extracellular domains of connexins (E1 and E2) play an important part in both mediating hemichannel docking and regulating voltage gating of the channel [36-38]. They provide the strong interaction between the two hemichannels, ensure the formation of an intercellular channel with no leakage of current and molecules to the extracellular environment [39]. The process of the formation of intercellular channels is dependent on the connexins expressed, and the second extracellular domain plays a decisive heterotypic compatibility between connexins [40]. In our study, Polyphen data shows that the mutation in *GJA3* is likely to be damaged. Although the activity of the P187S mutation identified in our study on the connexin 46 has not been immediately certificated, we presume that this mutation would result in an incorrect association of connexins and thus change the function of endogenous wild-type connexins in the affected individuals. Similarly, the mutation (P187L) in connexin 46 reported by Rees et al. [34], in 2000, which also localizes to the extracellular domain (E2) of the connexin 46 protein, strongly indicates an association of C560T with congenital zonular pulverulent cataracts.

The cataract phenotypes of *GJA3* mutations share genotype-phenotype similarities to some extent. Most of these were described as nuclear or zonular pulverulent types. Thus far, 16 mutations in *GJA3* have been reported to be associated with congenital cataracts in humans (Table 2). The mutation (P187L) in GJA3 described a Caucasian family with congenital cataracts, presenting with zonular pulverulent cataracts phenotype [34]. In our study, nuclear pulverulent cataracts (Figure 2) were fully exhibited in all the affected individuals in this Chinese family.

**Table 2:** Mutations in human connexin 46 (GJA3).

**Amino acid change**	**Location**	**Cataract type**	**Family origin**	**Reference**
D3Y	NH_2_-terminal cytoplasmic loop	Zonular pulverulent	Hispanic	[41]
L11S	NH_2_-terminal cytoplasmic loop	Ant-egg	Danish	[32]
V28M	First transmembrane domain	variable	Indian	[42]
F32L	First transmembrane domain	Nuclear pulverulent	Chinese	[43]
R33L	First transmembrane domain	Embryonal nuclear granular	Indian	[28]
V44M	First extracellular loop	Nuclear	Chinese	[44]
W45S	First extracellular loop	Nuclear	Chinese	[45]
P59L	First extracellular loop	Nuclear punctate	American	[46]
N63S	First extracellular loop	Zonular pulverulent	Caucasian	[47]
R76G	First extracellular loop	Total	Indian	[42]
R76H	First extracellular loop	Nuclear pulverulent	Australian	[48]
T87M	Second transmembrane domain	Pearl box	Indian	[49]
P187L	Second extracellular loop	Zonular pulverulent	Caucasian	[34]
P187S	Second extracellular loop	Nuclear pulverulent	Chinese	Present study
N188T	Second extracellular loop	Nuclear pulverulent	Chinese	[35]
S380fs	COOH-terminal cytoplasmic loop	Zonular pulverulent	Caucasian	[46]

Reported mutations in GJA3 associated with different congenital cataract phenotypes in different families.

In conclusion, we identified a novel mutation (P187S) in *GJA3* associated with autosomal dominant nuclear pulverulent cataracts in a Chinese family. As the first report to relate the P187S mutation in *GJA3*, it expands the mutation spectrum of *GJA3* in terms of congenital cataracts.
